# Modeling of Biomechanical and Functional Parameters of Hydrogel–Cell Composites Fabricated by 3D Bioprinting Using AI-Supported Approach

**DOI:** 10.3390/ma19081637

**Published:** 2026-04-19

**Authors:** Izabela Rojek, Maciej Gniadek, Jakub Kopowski, Tomasz Kloskowski, Dariusz Mikołajewski

**Affiliations:** 1 Faculty of Computer Science, Kazimierz Wielki University, 85-064 Bydgoszcz, Poland; maciej.gniadek@ukw.edu.pl (M.G.); jakub.kopowski@ukw.edu.pl (J.K.); dariusz.mikolajewski@ukw.edu.pl (D.M.); 2 Chair of Urology and Andrology, Department of Regenerative Medicine, Collegium Medicum, Nicolaus Copernicus University, 85-067 Bydgoszcz, Poland; tomasz.kloskowski@cm.umk.pl; 3 Institute of Advanced Studies, Nicolaus Copernicus University, Wileńska 4, 87-100 Toruń, Poland

**Keywords:** artificial intelligence, modeling, 3D bioprinting, hydrogel–cell composites, cross-linking kinetics, geometric stability, cell viability and function, simulation-based optimization, nonlinear parameter interactions, biofabrication processes

## Abstract

**Highlights:**

- AI-supported simulation framework for modeling hydrogel–cell composites in 3D bioprinting;
- Impact of cross-linking techniques and kinetics on mechanical strength and shape fixation;
- Time-dependent geometric stability of printed constructs;
- Cell-related constraints, including exposure to non-cross-linked matrices;
- Nonlinear relationships between printing parameters, material properties, and biological factors;
- Reliance on lowering time and material costs;
- Predictive assessment of biomechanical behavior prior to experimental validation.

**Abstract:**

3D bioprinting of hydrogel–cell composites requires simultaneous consideration of the biomechanical properties of the printed structures, the construct’s geometric stability, and conditions conducive to cell survival and function. Hydrogel cross-linking techniques and their kinetics play a key role in this process, determining the time of shape fixation, the mechanical strength of the structures, and the mechanical environment in which the cells are located immediately after printing. The relationships between bioprinting parameters, material properties, cross-linking strategies, and the presence of cells are highly nonlinear and often investigated through trial and error, leading to significant time and material costs. This paper proposes an approach based on artificial intelligence-assisted simulation, focusing on computer modeling of the biomechanical and functional parameters of hydrogel–cell composites produced by 3D bioprinting. The methodology is based on data generated from computer simulations and allows for analysis of the impact of printing parameters and different cross-linking strategies on mechanical strength, time-dependent geometric stability, and limitations related to cellular function, including exposure time to non-cross-linked matrices. The use of artificial intelligence methods allows for the integration of simulation results and predictive assessment of material behavior, providing a basis for future optimization of bioprinting parameters and process costs prior to experimental validation.

## 1. Introduction

3D printing as a tool for rapid prototyping and low-volume production has been on the market for over three decades. In recent years, with the dynamic development of tissue engineering and the growing interdisciplinarity of research spanning mechanical and biomedical engineering, biotechnology, and medicine, with significant support from IT tools, additive techniques have begun to be adapted for biological applications, collectively known as 3D bioprinting. However, this adaptation is not simply a transfer of experience from classic polymer printing, as the introduction of living cells into the material’s structure fundamentally changes the nature of design and process constraints. Unlike printing polymers, ceramics, or semi-liquids, bioprinting involves a biological-material system in which cells must remain viable and have access to oxygen and nutrients. In classic additive techniques, the main constraints revolve around flow parameters, viscosity, temperature stability, and environmental control, and time is not a critical factor for material degradation. In bioprinting, however, time, mass transfer, and physicochemical conditions directly impact cell survival and function, introducing an additional design dimension. This means that standard design methods and geometry conversion tools from a CAD model to a printer-readable format fail to account for a number of biological constraints, such as oxygen diffusion, cell residence time in bioink, or the local stability of the cellular environment. At the same time, the economic and organizational dimensions of the process are crucial. Unlike polymer printing, where the material cost is relatively low, in bioprinting, the value of the biological component—cell lines, hydrogels, and culture reagents—is high, and each failed attempt means not only financial losses but also the need to reculture the cells and extend the entire research cycle. From a clinical perspective, design errors can also translate into limited, timely availability of therapies. This complexity indicates the need for an approach that goes beyond classic slicer tools, which focus primarily on geometric aspects and extrusion parameters. Although models describing mass transport, material mechanics, and cross-linking kinetics are available in the literature, they typically function as standalone solutions and are not directly coupled to the design process. Therefore, what is lacking is not only their integration but also a coherent conceptual framework that allows for the simultaneous consideration of biological, material, and process aspects in the context of geometry generation.

Designing a construct in bioprinting should not be considered solely as a shape problem, but rather as a problem intertwined with mass transport, process parameters, and the mechanism of structure fixation. Some materials gradually cross-link over time, while others require the use of external factors such as light (e.g., UV), photosensitizers (e.g., riboflavin), or natural cross-linking agents (e.g., genipin). The fixation mechanism influences both the construct’s geometric stability—including the possibility of designing structures with microchannels—and the cellular environment, for example, by generating internal stresses or exposure to reactive factors. The nature of the problem being analyzed is nonlinear and multiparametric, making it difficult to solve based on isolated optimization of single variables.

The purpose of this publication is not to present a finished computational tool, but to outline a conceptual framework for its future development, discuss its possible architecture, and identify directions for further research. The proposed approach involves integrating mathematical and numerical models with the geometry preparation process, and potentially utilizing artificial intelligence (AI) methods for multiparametric analysis, process configuration optimization, and hazard identification, such as the risk of cell hypoxia or loss of construct stability. The ultimate vision is to create an analytical environment enabling the assessment of bioprinting project feasibility at the geometric model stage, which could reduce the number of experimental trials and their associated costs.

## 2. State of the Art

In extrusion 3D bioprinting, the process parameters determine the mechanical loads of the cells in the nozzle, which translates into both viability and mechanotransduction response as well as changes in gene expression [1,2,3,4]. Most often, shear stress is indicated as the main damage mechanism, for which a maximum at the nozzle wall and a decrease in viability with increasing τ have been described, with nonlinearity depending on the cell type and different sensitivity thresholds [1,4,5,6]. In practice, this load is controlled by process parameters: extrusion pressure is usually associated with the strongest decrease in lifetime (nonlinear increase in τ), while nozzle diameter and geometry are associated with modulation of wall shear stress, stress distribution and time of passage through the nozzle [2,3,5,6,7,8]. It was also emphasized that, in addition to shear, the extensional component in the constriction zone may be important, for which damage thresholds were reported and a potentially greater destructiveness than “pure” shear was suggested [1,3,8]. Since cellular damage depends not only on the stress level but also on the exposure time (residence time), the predictor τ × t was used in many approaches [1,3,4,6,7,8]. Experimentally, at higher loads, an increase in cell damage and stress indicators was reported, including changes in morphology and cytoskeleton, increased membrane permeability, and other markers of the stress response, while a low percentage of early apoptosis markers was reported immediately after printing in some conditions [2,3,4,5,6,7]. In parallel, gene expression changes after printing at higher stresses (stress genes and ECM and differentiation-related markers) were demonstrated, linking them to mechano-dependent signaling and potential influence on the differentiation direction, especially in MSCs, and adaptation/preconditioning concepts were described to increase resistance to subsequent high shear [1,2,3,4,7]. Cellular damage was most often modeled as a function of τ and t, determining stresses from rheological assumptions or using empirical regression models that linked process parameters involving living, damaged and dead cells [1,3,5,6,8]. The indicated limitations included evaluation only immediately after printing, lack of long-term functional evaluation or gene expression analysis, and model simplifications, including omission of cell–cell/cell–bioink interactions, lack of full CFD, and failure to consider the extensional component [3,4,5,6,7,8].

The “safe time” of cell residence in non-crosslinked bioink is most often determined indirectly in studies, mainly by observing damage during extrusion and assessing cell stability in the cartridge on a scale of approximately 1 h, and less frequently as a full dependence of viability on time in the bioink itself [9,10]. It was emphasized that the non-crosslinked polymer solution does not necessarily have to have a protective effect, because the protective effect was rather associated with mechanical “gelling” and crosslinking, and the sharp loss of viability during injection/extrusion was primarily attributed to the flow conditions [11]. In practice, 1 h of cell storage in selected bioinks is assessed without major problems with settling, and the sedimentation test in this horizon is used as a comparative metric for bioinks [9]. For longer times, the accompanying conditions proved to be critical, especially exposure to the photoinitiator and dehydration in air, which justifies minimizing the preparation and printing time and controlling the environment as a “technological limit” [9,12]. After fixation, with appropriate selection of printing conditions, no significant decrease in viability was reported in the short term after printing [13]. At the same time, no complete, quantitative “viability vs. time in uncrosslinked bio-ink” curves were identified for the horizon of many hours or a day in a single system, which indicates a data gap [9,10,11,12,13].

In bioinert, low-adhesive hydrogels, cell death is mainly due to the lack of adhesion signals leading to anoikis and limitations in mass transport (oxygen, nutrients, metabolites), while the effect of extrusion itself may be secondary depending on the model [14,15,16]. For adherent cells, the key mechanism of anoikis is associated with the loss of integrin signaling and activation of apoptotic pathways, including the FAK/ILK/Shc→PI3K/Akt axis, disruption of ERK signaling, and activation of the mitochondrial apoptotic pathway involving BH3-only proteins (e.g., Bim, Bmf), cytochrome c release, and caspase activation, with possible involvement of the JNK axis and elements related to caspase-8 and death receptors [14,17,18]. In bioprinting data, the decrease in viability was strongly dependent on the incubation time in bioink, and mortality was associated with anoikis and malnutrition resulting from limited medium availability. However, anoikis does not have to be immediate and its kinetics depend on the cell type and conditions [14,17]. The influence of cell density has also been emphasized, with higher density potentially accelerating death, which has been linked to both signaling from dying cells and coupling with transport limitations [15,16,17]. The second key mechanism is transport limitations in larger volumes and at greater thickness, leading to hypoxia with oxygen gradients and a decrease in the concentration in the center, and to a transition from apoptosis to necrosis in profound hypoxia, which may be accompanied by glucose and trophic factor deprivation, metabolite accumulation, local acidification, and secondary mitochondrial damage [15,16]. Under bioinert conditions, a cellular stress response component and inflammatory markers were also observed, indicating that the non-native microenvironment can induce biological changes even without the dominant contribution of shear stress [14]. Interpretation is complicated by cellular heterogeneity, as cancer cells may exhibit resistance to anoikis and survive, proliferate, and form spheroids in a non-adhesive environment, which affects comparisons between cell lines and types [17]. Critically, the need to separate the contributions of non-adhesion, transport limitations, and process effects (mixing and short-term mechanical stress) has been pointed out, as in some models, incubation time and non-adhesion outweigh the printing stage itself [14,15].

The “maximum oxygen diffusion distance” is practically defined as the distance from the oxygen source after which biologically significant decreases in pO_2_ begin to dominate in the construct core, with threshold values of approximately 100–150 µm and a design range of 100–200 µm most often cited [19]. This principle is compared to the in vivo physiological rule, according to which cell survival requires maintaining a distance of approximately 200 µm from a vessel or other oxygen source, and exceeding these distances is associated with the development of hypoxia in the center, decreased viability during culture, and the risk of a necrotic core at greater thicknesses [19,20]. The distance itself is not a material constant, as it depends on the oxygen diffusivity in the hydrogel and the rate of consumption by cells, with a strong dependence on cell density, and it has been pointed out that the oxygen diffusivity in hydrogels may be significantly lower than in water, which narrows the permissible transport distances [19]. To move from the 100–200 µm heuristic to quantitative relationships, reaction-diffusion modeling (often 1D) is used, including diffusion and oxygen consumption, including Michaelis–Menten kinetics, and in one of the approaches, examples of oxygen diffusivity in gels with cells (D*G) of the order of 1.01 × 10^−5^ cm^2^/s for rBM, 1.18 × 10^−5^ cm^2^/s for dense FIB, 1.25 × 10^−5^ cm^2^/s for dense COLI and 1.46 × 10^−5^ cm^2^/s for sparse COLI were given, and hmax and qo,max were projected relative to the normoxia thresholds of 50 mmHg and hypoxia thresholds of 10 mmHg [21]. For example, with the pO_2_ criterion assessed at half thickness after 1 week, hmax of 1.06–1.35 mm for rBM, 2.46–3.2 mm for dense COLI and 1.6–2.08 mm for dense FIB was reported, with corresponding qo,max in the ranges of 0.14–0.22 × 10^6^ cell/mL (rBM), 0.76–1.27 × 10^6^ cell/mL (dense COLI) and 0.14–0.54 × 10^6^ cell/mL (dense FIB), which shows that the permissible thickness and cell density are strongly material-dependent [21]. In another example, for alginate, it was shown that at high cell densities, the required thicknesses can drop below 1 mm, while for 2% alginate and η = 0.9, approximately 0.86 mm was given in the considered scenario [22]. Experimental mapping of oxygen gradients using microsensors (phase fluorimetry) allowed for determining the O_2_ profile and indicated the transition to hypoxia within the cellular region and the boundary of this region at a radius of 850 µm, which illustrates the core’s entry into hypoxia on a scale of hundreds of µm for a given geometry and cell density [23]. The design conclusion is that without perfusion and internal channels, the geometry, cell density, and material should keep cells at distances of 100–200 µm from the oxygen source, and for larger thicknesses, strategies to shorten the distance by introducing oxygen sources inside the construct are needed [19,20].

Increasing the construct thickness lengthens the oxygen diffusion path and promotes the formation of steep pO_2_ gradients, which, in conditions without convective mass transport, limits cell viability in central areas [24,25]. The literature emphasizes the maintenance of cell survival and function mainly within a distance of approximately 200 µm from the tissue surface or from the perfusion channel, which is the local oxygen source, and in static cultures, a decrease in central oxygen levels to 0% after approximately 5 days has even been reported [20,25,26]. In the cardiac construct model (approximately 1.84 mm thick), a decrease in oxygen from approximately 176 µM to approximately 22 µM along the thickness axis was measured, accompanied by a decrease in viability with depth to values of 15–5% below 1000 µm and maintaining values close to physiological values only in the outer layer of approximately 128 µm; It was also indicated that at oxygen concentrations above 120 µM, the viability was at least about 60%, while below 70 µM at a depth of about 1000 µm it dropped below 20% [24]. In thick 9 × 9 × 4 mm^3^ constructs, it was shown that without perfusion, the oxygen concentration decreased from top to bottom independently of the microchannels, while perfusion eliminated the gradient and maintained values close to the level in the medium (~18%), and after switching off the perfusion, the oxygen in the center decreased exponentially from about 17% to about 12% in <20 min and returned to about 17% in 13–16 min after resuming the flow [20]. Accordingly, the viability in the center decreased in the non-perfused variants on days 7 and 14, while in the perfused constructs it increased to approximately 80% on day 14, which was associated with the provision of mass transport in thick structures [20]. It was also indicated that microarchitecture may shift the limit of viability decline, because in collagen constructs with medium access on one side, the viability dropped below 85% already at approximately 200 µm in the solid construct, and at 75% porosity it remained above 85% up to approximately 1 mm, and in larger systems, the combination of 75% porosity and perfusion increased the radius of viability maintenance > 75% to approximately 4 mm (with a significant improvement reaching approximately 5 mm) [26]. Viability heterogeneity in the thickness axis was also observed in systems with bioink between thermoplastic filaments, where more dead cells occurred in the lower half, which was attributed to hypoxia-like conditions at the bottom, limited exchange of medium and oxygen, material factors, and degradation of the gelatin component [27]. The severity of hypoxia depends on cell density and metabolic activity, and changes in oxygenation may trigger HIF-1α-regulated responses; in comparison to porous and solid constructs, a higher expression of hypoxia markers was demonstrated in solid constructs and no detection of HIF-1α in porous constructs after longer culture [25,26].

Design strategies are used because passive oxygen diffusion limits volumetric oxygenation and requires maintaining cells at a short distance from the oxygen source (capillary/channel/perfusion zone), typically in the order of 100–200 µm [28,29,30,31]. The most frequently described approach is the introduction of perfusable microchannels to shorten the diffusion path and simultaneously enable the inflow of oxygen and nutrients and the removal of metabolites. The channels are fabricated using, among others, templates or sacrificial inks (e.g., Pluronic F127, gelatin, agarose, carbohydrate glass), which, after removal, leave a perfusable network [28,29,30,31]. It is emphasized that not only the creation of an empty channel is critical, but also its endothelialization as a step towards functional vascularization, including in situ endothelialization approaches, which improve the uniformity of seeding versus post-seeding [28,31,32]. Perfusion is crucial, as the transition to convective-diffusion transport significantly improves oxygenation and survival in thick constructs, and is implemented in both bioreactor and microfluidic systems, where exchange occurs at the interface between flow in the channel and diffusion into the matrix [29,30,31,32]. In parallel, biological strategies are being developed, such as prevascularization with endothelial cells (often with supporting cells) to shorten the critical diffusion-dependent period after implantation, and the promotion of angiogenesis by proangiogenic factors and matrix functionalization (factor binding and controlled release systems) to accelerate host vessel ingrowth and stabilize the network [30,31,33]. Translational approaches also indicate bridging approaches, including oxygen-providing materials and tactics that suppress cellular metabolism to reduce the risk of necrosis before full perfusion or blood supply is achieved [31]. Limitations of these methods result, among others, from the extrusion printing resolution (tens–hundreds of µm), which hinders the mapping of 10–20 µm capillaries, as well as from the multi-step nature and risks of cytotoxicity of sacrificial materials and difficulty in removing the template in large volumes [29,31]. In practice, the design comes down to controlling the maximum cell-channel/vessel distance, the geometry and connectivity of the channel network, flow conditions ensuring transport without undesirable shear, and the biological readiness of the construct for rapid vascularization [29,30,31,32,33].

The choice of hydrogel cross-linking strategy is critical because it simultaneously determines the stability and fidelity of the construct’s shape, mechanical parameters, and cell microenvironment conditions, including mass transport and process exposures [10,34,35]. Literature distinguishes physical cross-linking (thermal, ionic, and electrostatic), chemical covalent cross-linking (including UV/visible light photopolymerization, also in situ during extrusion), and hybrid and reinforcing strategies, such as dual-crosslinking, interpenetrating networks, self-healing supramolecular systems, nanocomposites, and thermoplastic micro-reinforcements [10,34,35,36]. Mechanics are coupled with the degree of cross-linking and polymer content, as increasing network density usually increases stiffness and stability, but may limit porosity and permeability, impairing nutrition and oxygenation, as well as long-term cell function [10,35]. Therefore, the separation of mechanical control from diffusion is emphasized, e.g., by modifying the degree of substitution of reactive groups, which allowed for obtaining a wide range of moduli of 1.8–63 kPa without significant changes in diffusion indices in some variants [37]. In photocuring, attention is drawn to the existence of a process window: too weak curing does not stabilize the structure, while too strong can hinder subsequent fiber bonding, while UV and photoinitiators may reduce viability depending on the dose and concentration, which justifies the optimization of time and intensity, as well as the selection of the initiator, and the preference for visible light as less biologically risky [10,34,36]. In the context of process stress, it has been pointed out that solutions enabling printing from lower viscosity or improving the flow profile can limit cell damage, and in one comparison, pre-crosslinking before extrusion was associated with a viability of about 47%, while an in situ approach during extrusion resulted in about 95–96% post-printing viability and maintained high viability after culturing [36]. In ionic systems, the critical parameters are the type and concentration of ions and the method of their application, as they affect stiffness, spreading, and stability, while too rapid gelation can impair geometric stability due to inhomogeneous curing; at the same time, despite the lack of UV exposure, excess ions and exposure time can reduce viability [34]. Because random networks tend to be brittle due to limited energy dissipation, reinforcing approaches are being developed (penetrating networks, supramolecular sacrificial bonds, nanoparticles, thermoplastic micro-reinforcements) to improve mechanical parameters without simply increasing the network density [10,35]. The cell response to stiffness depends on the availability of adhesion sites and mechanotransduction, therefore cross-linking strategies are combined with the control of adhesion ligands and network degradation, which in HA-based systems was associated with the selection of RGD-type adhesion motifs and the choice of a degradable or non-degradable crosslinker [35,36]. The degree of cross-linking and the method of its achievement remain a design variable that simultaneously controls mechanics, mass transport, and cell biology, which implies the need for multi-criteria optimization and modeling frameworks that simultaneously consider mechanics, diffusion, and process-dependent viability metrics [10,35,37].

Shape fidelity in extrusion bioprinting depends on whether the filament achieves load-bearing capacity sufficiently quickly after deposition, before gravitational deflection, viscoelastic relaxation, and surface tension-related pore spreading and closure occur [38]. Gelation kinetics is practically defined as the time to reach the effective yield stress or critical G′ stabilizing the filament after the cessation of shear (recovery kinetics), and quantitatively, tgel is sometimes defined as the intersection point of G′ and G″, strongly dependent on temperature and concentration [38,39,40]. The actual fixation time after deposition Δtgel relative to the interlayer time Δtlayer is crucial for the process, as a sufficiently short Δtgel enables multilayer stability and acceptable printability (the criterion Pr > 0.9 was indicated, among others), while both too slow and too fast gelation reduces fidelity (under-gelation: fusion and loss of stability; proper-gelation: continuous filaments and regular mesh; over-gelation: jet breakage and irregularity) [38]. At the geometrical level, critical phenomena include filament collapse (Z axis) and fusion and pore closure (XY plane), which are particularly sensitive to the early seconds after deposition, because deformations occur mainly in the first ~20 s, and increasing yield stress limits both the deflection of the spans and the fusion of adjacent lines [39]. The limitations of slow gelation can be overcome by immediate support (e.g., FRESH) or the use of jammed microgels, where rapid yield stress ensures fidelity, and secondary cross-linking is mainly responsible for mechanical durability [41,42]. In photoinitiated systems, the gelation rate controlled by exposure parameters translates into geometric stability, as too short an exposure promotes deformation, while too long can cause over-hardening and cracks, with the importance of cross-linking uniformity across the filament cross-section [43]. The review emphasizes the advantage of two-step strategies combining rapid geometric stabilization with secondary reinforcement, while the conflict with biological requirements must be taken into account, as intense and rapid cross-linking may worsen cell conditions [40].

Thermoresponsive hydrogels enable operation in the sol state at lower temperatures, which facilitates cell mixing and loading, and upon increasing the temperature, they transform into the gel state, promoting dimensional stability during deposition [44]. Since a reversible physical network alone is often insufficient for long-term stability and mechanical properties, additional chemical fixation is often used, e.g., dual-crosslinking systems combining thermogel with photopolymerization [44,45]. Chemical covalent crosslinking produces an irreversible and more stable network after printing (e.g., photochemistry, click chemistry, enzymatic crosslinking), which is preferred for longer culture times or higher loadings [44,45]. In the thermo + photo model, it was shown that dual cross-linking allowed for obtaining controlled architecture and stability (porous structures up to ~0.6 cm in height), and photopolymerization strengthened the physical network, increasing G′ approximately hundred-fold (from 0.35 to 36 kPa) and maintaining high chondrocyte viability (94 ± 3% after 1 day and 85 ± 7% after 3 days) with the declaration of no adverse UV effect under these conditions [44]. At the same time, the review emphasized that in photochemistry the photoinitiator, intensity and time of exposure are crucial, because long times and high intensities may be associated with DNA damage, and the UV range of 290–320 nm is indicated as unsuitable for bioinks with cells, which justifies the development of approaches based on visible light [45].

The modeling of oxygen and nutrient transport in bioprinted constructs is most often based on mass balance and Fick’s law, leading to diffusion equations (often 1D) with typical boundary conditions, and then to the diffusion-metabolism approach by adding a consumption term, including quasi-steady-state approximation and solutions in planar, cylindrical and spherical geometries [46]. In this framework, orders of magnitude of diffusion coefficients in hydrogels/polymers of the order of 10^−9^–10^−10^ m^2^/s (oxygen closer to 10^−9^, glucose closer to 10^−10^) are considered and design metrics of the type T_max/R_max (maximum allowable thickness or distance to the source) are derived, taking into account that diffusion from two sides in planar geometry increases the allowable T_max relative to the one-sided case, and partial bypassing of diffusion constraints is also considered by layered regionalization of metabolism [46]. For full 3D geometries, digital twin continuum models are used, based on coupled PDEs for oxygen and glucose and a non-stationary equation for cell density, where consumption is described by Michaelis–Menten kinetics and cell growth by the Monod equation, with Robin conditions at the medium–construct interface and FEM solutions (e.g., FEniCS (open source, https://fenicsproject.org/, accessed on 1 April 2026), also Matlab R2026a (MathWorks Inc., Natick, MA, USA)), while solving linear systems using the GMRES method with ILU [47]. Such simulations can predict the formation of a necrotic core when oxygen drops below the constant K_m (MM for survival), and the introduction of channels in the construct can eliminate necrotic areas by redistributing the availability of substances [47]. For perfusion environments, a class of flow–transport models is described combining cellular automata for population dynamics, Lattice Boltzmann for hydrodynamics in the scaffold (e.g., D3Q19 with bounce-back), and the convection–diffusion equation for oxygen with Michaelis–Menten consumption, where the steady-state velocity field is first determined and then used to compare static and perfusion cultures [48]. Design approaches also use COMSOL6.4 (COMSOL Inc., Burlington, MA, USA) to predict oxygen gradients and select vascular network geometry in CAD, modeling convective-diffusion flux (with u = 0 in the aggregate volume), Michaelis–Menten wear (e.g., N_0_ = 0.012 μmol/10^6^ cells/h, K_MM = 0.011 mol/m^3^) and a functionality threshold of 0.04 mM (4% O_2_), with parametric analyses of spacing (1.5–9 mm) and wall permeability (e.g., silicone vs. FEP) in a model–design loop related to experimental viability assessment [49]. For implantation and filling situations, CFD in ANSYS Fluent 2026R1 (Canonsburg, PA, USA) is used, combining the VOF for the blood-air system with species transport of oxygen and glucose, with the continuity and Navier–Stokes equations and Fick’s law for the diffusion flux, taking into account the non-Newtonian power-law rheology and given boundary conditions of concentrations and inlet velocities, with simulation up to 2000 s as a model assumption corresponding to the beginning of solidification, and qualitative validation based on the comparison of oxygen and glucose distributions with the distribution of new bone tissue in vivo (oxygen trend more convergent than glucose trend) [50]. Hybrid agent-based approaches are being developed separately, e.g., with the Cellular Potts Model, where the concentration fields are described by a parabolic PDE (∂_t C = D∂_iiC + S), consumption is introduced by a cell type-dependent source term and consumption parameterization (with reference to the literature), and cell dynamics and death depend stochastically on concentrations and oxygenation, which is analyzed in the context of core hypoxia and sensitivity to oxygen boundary conditions [46,51].

Finite element methods are used to estimate the local mechanical state in cell-supporting hydrogels (stresses and microenvironment measures), which cannot be reliably imposed and measured in situ in 3D, and therefore are reconstructed computationally [52,53]. Scaffold-hydrogel models use a repeating unit with an ROI corresponding to the cell volume and analyze, for example, strain energy as an indicator of the mechanical microenvironment, with the fibers described as linear elastic and the hydrogel as a compressible hyperelastic material (e.g., compressible reduced polynomial), calibrated on data from compression tests (free and constrained), with model selection for fit and numerical stability, and with the need to account for large deformations [52,54]. FEM is also used as a “virtual test” to calibrate the properties of protein hydrogels under uniaxial loading and stress relaxation, combining hyperelasticity with viscoelasticity described by the generalized Maxwell model (Prony series) and selecting parameters with control of material stability (Drucker’s criterion) [55]. In micromechanical variants, cells are explicitly modeled as a composite component (hyperelastic hydrogel, linearly elastic cells with given E and ν), and heterogeneity is represented by random distribution of cells generated by a Python 3.14 (open source, Python Software Foundation) script and assigning different material properties to the elements, e.g., in a strand (cylinder) geometry with boundary conditions and large displacement enabled [54]. Control of mesh and convergence (mesh refinement, ROI densification) and sensitivity to boundary conditions are crucial, as modifications to out-of-plane constraints can radically change the response in the ROI and require interpretation as an idealization of in vitro or in vivo conditions [52]. A separate class is coupled mechanics-fluid modeling in the poroelastic approach (two-phase continuum, fluid pressure + solid phase response, deformation-dependent permeability), calibrated on progressive stress-relaxation tests in unconfined compression, implemented, e.g., in FEBio (quarter cylinder model, 20-node hexa elements) with Levenberg–Marquardt optimization and verification on an independent cyclic compression test [53]. For bioink strands, validation was reported by comparing stress–strain curves from the tensile experiment (alginate without and with cells) and comparing the stress and strain limit values, with the resource-intensive calculations of microscale models taking into account heterogeneity being indicated as a barrier [54].

Current simulation approaches have limitations in fully capturing the dynamic, multi-scale nature of bioprinting, including real-time cell–matrix interactions, variability of bioink properties during the process, and construct maturation [56]. In practice, optimization is problematic due to the large number of parameters influencing the result, which necessitates costly iterative tuning. Additionally, some “optimal” geometries from simulations are sometimes infeasible in printing (printability constraints), which exacerbates trial-and-error [56]. A key limitation is the reliable modeling of hydrogel mechanics (nonlinearity, heterogeneity, time dependence) and the choice of constitutive law: overly simple models do not capture temporal effects or fluid-solid couplings, while more accurate models require more calibration data (sometimes also inverse modeling) and significantly increase computational costs [56]. Validation is hampered by low rheological stability during fabrication, limited usefulness of standard mechanical tests for very soft constructs, and the need for in situ measurements and imaging, as relaxation during and after deposition can cause a geometry mismatch between simulation and reality [56]. Furthermore, the dynamic nature of the process (cooling, heating, extrusion, cross-linking) means time- and temperature-dependent properties, which increases complexity and computational burden, while failure to account for biological maturation processes (swelling, diffusion, proliferation, ECM deposition) can make models useful only for the early phase and unreliable after maturation [56]. Practical limitations also apply to standardization and evaluation of printability: the lack of uniform metrics complicates comparisons, and printing trials are time- and material-intensive, with bio-inks with cells additionally limited by the time after mixing and the risk of decreased viability [57]. This can result in printing under suboptimal conditions, with reduced fidelity and difficulty in separating the effects of material, printing parameters, and cell addition, which can dilute the system and reduce viscosity [57]. Simulation can also be limited to shear forces in the nozzle, while construct stability assumes that the printout complies with CAD, which is rarely the case in hydrogels. Therefore, the need to simulate dispensing and deposition outside the nozzle is indicated, taking into account additional shear and viscoelastic stresses [57]. In macroscale flow and transport models, the Navier–Stokes parameterization for viscoelastic fluids and models of transport and response of biochemical factors is problematic, and in two-phase models, maintaining a sharp boundary, correctly calculating surface tension forces, and limiting numerical errors (e.g., mass loss/gain in the level-set) is difficult, with additional limitations of interface tracking methods (front-tracking/IBM/CSF) [57]. Limitations of mesoscale approaches have also been pointed out, where cellular automata are useful mainly for equilibrium states, implementations are often limited to asynchronous updating, and in CPD difficult parameterization remains a problem [57].

AI/ML is presented in reviews as a way to reduce trial-and-error in the selection of bioprinting parameters by training models on process data and print results and using them to predict quality and select settings [58,59,60,61]. In the Quality by Design approach, models map CMA and CPP to CQA, supporting the definition of design space and the design-before-print stage, where quality metrics (printability, shape fidelity, resolution, filament width/morphology, as well as CQAs such as maximum shear stress and cell viability) are predicted based on process parameters and material characteristics [59,60,61,62]. Regression and classification models (including GP/GPR, RF, SVM, and neural networks, including MLP) are used, and process maps and phase diagrams of the parameter space are constructed to facilitate the selection of settings in areas of stable printing. A hierarchical workflow has also been reported, in which printability increased from 85.2% to 98% [59]. Optimization is often iterative: Bayesian optimization and active learning select subsequent experiments/settings based on previous results, and the closed-loop approach involves layer-by-layer analysis of monitoring data, detection of deviations, and generation of CMA/CPP corrections, which is sometimes combined with computer vision and robotic bioprinting platforms [58,59,61,62]. Reinforcement learning for control in dynamic scenarios, combining ML models with heuristic search, and multi-objective optimization (Pareto) for trade-offs between requirements, as well as inverse design/property-fingerprint, where the model starts with the required properties or CQAs and proposes parameters or a design, has also been cited in this context. The use of synthetic data for training error detection and geometry has also been mentioned [58,59,60,61,62]. Recent reviews further indicate that AI in 3D bioprinting can support not only material and quality prediction, but also printing-process modeling and optimization of printing parameters through process–structure–property relationships [63].

Prediction of mechanical properties of hydrogels in biofabrication utilizes data from simulations, mechanical tests, and metasets built from the literature, and the selection of ML methods depends on whether the target is single parameters (e.g., E, ν, G′, τy) or full material response curves [64,65,66,67]. In the FE→training set→surrogate approach for the BG–COL composite hydrogel, RVE microstructure images are generated, E and ν are determined in Abaqus, and then a CNN is trained to regress these properties from 2D images (200 × 200 px), reporting an accuracy of ~95% for E and ~83% for ν with a set of 2000 images, with the limitation of not taking into account the effect of collagen cross-linking [64]. The second trend maps the “printability window” from mechanical and rheological measures of bioinks, especially G′ and τy, and their relationship with process metrics (extrusion, shape fidelity), where G′ dominated for fidelity (~84.6% of explained share) and τy (~89.5%) for extrusion, and multiple regression equations (with collagen/HA/fibrin component interactions) were used to predict G′ and τy and to determine the normalized NV criterion (high G′, low τy) as the operational window [68]. In metadata approaches from the literature, classical supervised models are used to predict mechanical properties based on formulation and process features (with coding of categorical features), comparing, among others, AdaBoost, Gradient Boosting Regression, KNN, SVM, XGBoost, and MLP, with XGBoost selected as the leading model, with hyperparameter tuning (Optuna) and interpretation of feature contributions using the SHAP method [65]. A separate class of deep learning models are trained on synthetic data from mechanical models (e.g., SAW + constitutive description) to predict full nominal stress–stretch curves for single-network hydrogels (PAAm), where DNN/MLP operated on features describing the network, and 3D CNN on spatial data representing the network, with labels as a set of stress values for given stretches [66]. Similarly, in a DOE + DNN approach trained on mechanical test data for porous hydrogels (PVA/gelatin), samples were designed using the Box–Behnken method, and the feed-forward network learned to reproduce the stress–strain profile using PVA, gelatin, and crosslinker concentrations and test duration as inputs, and stress and strain values as outputs, when implemented in MATLAB and trained on >30,000 data points [67].

In the Quality by Design approach, the integration of biomechanical and biological constraints is described by jointly defining CQAs as physical, chemical, and biological properties, distinguishing between forming-based CQAs (printability predicted from rheology and gelation kinetics) and function-based CQAs encompassing transport, mechanics, and biology, where geometry and microstructure simultaneously influence mechanical and biological properties, and biology is considered from the tissue level to the cell and gene expression level [59]. Bioink/formulation is treated as CMA, process parameters as CPP, and design space as maps/windows of stable CMA–CPP combinations meeting CQAs, supplemented with risk assessment for the influence of variables and interactions, which shifts the optimization from a sequential approach to a joint optimization of mechanics and biology [59]. Since microstructural features important for stiffness are also critical for mass transport, integration is presented as combining ML with multi-objective (Pareto) optimization and inverse design approaches, where ML models map expected mechanical properties to the microstructure design, and the biological component is incorporated as an equal criterion by mapping microstructure to cell behavior [59]. At the same time, the limitations of biological data after bioprinting are pointed out as costly and time-consuming, and the need to combine ML with multi-scale modeling and spatiotemporal models of cell behavior for simultaneous prediction of transport, mechanics, and biological response evolution has been pointed out, with only preliminary attempts at such integration reported [59]. Integration is sometimes described as an AI-based systematic design approach encompassing the design of bioinks, structures and printing parameters, and curing conditions, as well as an extension to 4D bioprinting, where AI combines microstructure, spatial distribution of formulations, and external stimuli to obtain spatiotemporal mechanical response [59]. At the workflow level, integration is also presented as closed-loop control and a full preprinting–printing–postprinting chain, where ML supports bioink optimization, modeling/digital modeling, layer-by-layer monitoring, quality assessment, and iterative tuning (e.g., CNN for quality classification and stable process maps, active learning, Bayesian optimization), with the intention of supporting technical and biological aspects in parallel [59,69]. Examples have been indicated in which CQA includes both cell viability and process/structure metrics (e.g., filament diameter, pressure), and biomechanics was indirectly addressed through load prediction (e.g., maximum shear stress modeled with GP methods) to support CPP selection under biological constraints [59]. At the same time, in selected areas, e.g., modeling of the tumor microenvironment, it was emphasized that the literature directly integrating AI-driven methodologies with 3D bioprinting remains very limited, which was associated with the need for workflow standardization, multiomics data integration and clinical validation [69].

The analyzed sources described only partially integrated approaches that combine selected process elements but do not create a single, coherent framework encompassing hydrogel mechanics, oxygen diffusion in the construct, cross-linking kinetics, and cell viability simultaneously [6,70,71,72,73]. On the cross-linking side, the coupling of Ca^2+^ ion transport with cross-linking kinetics was demonstrated, where the degree of cross-linking α(x,t) controls the diffusivity D(α) and changes the prediction of the gelation front (the constant D overestimated the front width by ~20% and ~100 µm after 10 min), with validation based on a syringe weighing experiment and noted inconsistency in the error report (text ~6% vs. table 11.33% for D(α)). [6] In another approach, the cross-linking front in Alg–Gel–Hyal was described by the relation x_c = k√t (also in cylindrical geometry) and integrated with mechanics by tracking G′(t) and determining the cross-linked layer thickness r(t), and then extended to 3D simulations in Python (STL→voxelization→erosion), indicating processually that porous scaffolds shorten the bath time in CaCl_2_ compared to a solid body, which has consequences for the exposure time of bioink with cells [71]. On the flow and diffusion side of the nozzle, a core–sheath model was described in a microfluidic coaxial head, coupling steady-state laminar flow with diffusion (Navier–Stokes + Fick) to predict the composition at the cross-linking initiation site, without explicitly considering the polymerization itself, and the connection with biology was realized by viability tests as a function of irradiance and photoinitiator, and evaluation of glycerol cytotoxicity at the concentration predicted by the model (34% *v*/*v*) [73]. Process constraints were also integrated as proxies for biological constraints: CFD in OpenFOAM was used to calculate MSS for nozzle and bioink geometries (power law), with sensitivity analysis supported by Gaussian Process, with the constraint of a single-phase description without an explicit cell model, and analytical-rheological approaches combining filament width prediction and shear stress estimation with experimental validation and viability assessment [6,72]. Consequently, the integration remains fragmented: transport concerns Ca^2+^ or diffusion of components in the nozzle, and biology is mainly related to photocuring conditions, composition (e.g., glycerol), or shear stress, without fully incorporating oxygen diffusion constraints in the construct volume in the same framework [6,70,71,72,73].

The geometry and architecture of hydrogel trusses control the mechanical stability and compressive response through parameters such as cell unit size, strut diameter, and unidirectional cell scaling, which allow tuning the stiffness and anisotropy in shear and compression, because the truss mechanics arise from the work of beam elements and the mechanisms of compression, shear, bending, and buckling [74]. Increasing the cell unit size from 1.25 mm to 2.00 mm led to a strong decrease in moduli, described as a decrease of ~90% for apparent Young’s modulus and ~85% for shear modulus, while the change in strut diameter was even more critical, as a decrease in strut diameter from 300 µm to 200 µm was associated with a decrease in apparent shear modulus up to ~95%, and in the data, an increase in diameter from 200 to 300 µm corresponded to an increase in G′ of about 18× (from ~1.3 kPa to ~24 kPa) [74]. These effects were associated with volume fraction and porosity: with an increase in volume fraction from 0.12 to 0.29 and a decrease in porosity from 0.88 to 0.71, an increase in modulus was observed, indicating volume fraction as a key predictor of stiffness, and power-law relations and the Gibson–Ashby model were used to predict moduli based on this, which was found here to be quantitatively accurate for apparent Young’s modulus [74]. Scaling the unit in the X direction from 1.00× to 2.00× increased anisotropy in shear and compression (~3.1× and ~2.9×, respectively), while it was pointed out that the orientation of the printing process may introduce unintended anisotropy (modulus in the build direction larger than in the transverse direction) through layered curing and stair-stepping artifacts [74]. The effect of geometry was generalized by FE simulations in Abaqus/Standard (parameterization of unit cell size, strut diameter, and scaling) to predict apparent shear and Young’s modulus over a wider range of geometries than the tests, with the limitation that the simulations did not include the effects of build direction, differences between CAD and print, or swelling in water conditions, which limits transferability beyond ideal geometry [74]. In this work, “compressive strength” was treated operationally as the apparent Young’s modulus of unconfined compression determined from the linear section of the stress–strain curve up to 5% strain, rather than as the strength to failure [74].

Multi-criteria optimization in scaffold design is used because mechanical and biological requirements are conflicting: increasing stiffness usually comes at the expense of porosity, which worsens fluid transport and conditions for adhesion, growth, and vascularization [75]. In the case of bone scaffolds, the “biological” side is represented by metrics such as permeability, pore size, porosity, and specific surface area (Sv), with a pore size range of approximately 400–1000 µm indicated as favorable to avoiding blockage and providing space for vascularization, adhesion, and proliferation processes [75]. For example, the problem was formalized as a bi-objective maximization, where the effective Young’s modulus (Eeff) and permeability (k) were simultaneously maximized, with additional constraints on throat size (Pmin–Pmax), Sv > Svmin, structural integrity, and constant porosity ϕ = ϕ_0_, and the decision variables were the cell unit geometry parameters and pore radii (MFCC) or strut diameters and pore/layer adhesion parameters (OCS) [75]. First, a feasible design space was determined from the structural constraints and cell growth conditions, then the targets were calculated for all feasible combinations, and the search was performed using exhaustive search with a 50 µm discretization resulting from the accuracy of the Ultimaker S3 [75]. The pipeline combined analytical formulas for porosity and Sv with FEM for Eeff (automatic model generation in Abaqus using a Python script, small-strain loading, and determination of Eeff from force response) and CFD for k (DMEM flow, constant inlet velocity, determination of k from Darcy’s law based on pressure drop) [75]. After design calculations, a Pareto front in (E, k) space was constructed and representative solutions (maximum stiffness, maximum permeability, and compromise) were selected. The compromise was determined using the Utopia point method after normalization of targets and minimization of distance from the ideal point, and additionally, solutions were filtered with respect to the permeability range reported for human trabecular bone [75]. The results showed a stiffness–permeability trade-off, an inverse stiffness–porosity relationship, and that geometry alone (e.g., pore radii) can significantly tune both targets even at constant porosity, and the MFCC designs enabled high porosity with relatively high stiffness relative to OCS; selected scaffolds were fabricated in FDM and tested in compression, observing larger FEM–experiment discrepancies at very high porosities due to design and print mismatch for small features [75]. Limitations included simplification of loading to static uniaxial compression, dependence of permeability on DMEM viscosity, problems with FDM fabrication for porosity > 85%, and poor scalability of exhaustive search (genetic algorithms were suggested), and the biological component was limited to permeability/pore size/Sv without oxygen diffusion or cell viability models [75].

Typical oxygen diffusion distances in tissues are reported to be limited to approximately 100–150 µm, and for glucose, a range of approximately 5–200 µm is reported, which is associated with a decrease in survival when these limits are exceeded and with the observation that in static culture, areas more than 0.5–1 mm from the surface may contain only dead cells. Quantitatively, diffusion coefficients of small molecules in scaffolds of the order of 10^−9^–10^−10^ m^2^/s and variability of oxygen diffusion in biomaterials relative to water have also been cited [76]. In addition to microchannels and perfusion, photobiomodulation as a non-invasive method has been discussed as a strategy to alleviate transport limitations, with discussions of the “optical window,” dose, and light absorption and scattering in hydrogels [77].

Oxygenation in hydrogels was validated and measured both biologically, using hypoxia reporter cells (fluorescent UnaG reporter controlled by HIF-1α), and instrumentally, by non-invasive sensor-spot measurements with the Oxy-4 mini system (element diameter 3 mm) positioned in the gel with a printed holder, with oxygen limitations reported to be more pronounced in softer GelMAs allowing better cell spreading, and preparation conditions included GelMA 3.5–4.5% *w*/*v*, Irgacure 2959 0.2% *w*/*v*, temperature control at 33 °C with cooling to 25 °C, and a UV dose of 1.4 J/cm^2^ [76]. In Si-HPMC hydrogels, the oxygen diffusion coefficient was shown to decrease with increasing polymer concentration (from 3.4 × 10^−10^ to 2.4 × 10^−10^ m^2^/s for 1%→4% *w*/*v*), and in vitro culture it was noted that oxygen depletion occurred before glucose depletion, which supports the conclusion that oxygen is the main factor limiting survival; at the same time, a correlation between glucose parameters and the size of cross-linking nodes was indicated, in the absence of a similar correlation for oxygen, and validation schemes were used, including ranges of polymer concentrations and seeding densities, as well as Live/Dead assessment at time points [19].

On the transport modeling side, ready-made equations and analytical solutions of diffusion with metabolism were presented for planar, cylindrical and spherical geometries, together with formulas for the maximum thickness/size under given boundary conditions, diffusivity and consumption rate, comparing typical ranges of diffusion coefficients in tissues and hydrogels and typical ranges of C_0_ of oxygen in the medium, and as a partial bypass of the limits, regionalization of the metabolically active outer layer in spherical constructs was proposed [46]. The core–shell model uses a spherical reaction–diffusion description with Michaelis–Menten consumption in the core and no consumption in the shell, identifying size thresholds favorable for maintaining viability (core radius ~142 µm or less and shell radius < ~283 µm, as well as the case of ~100 µm core at low external oxygen concentration), with a numerical solution (shooting + RK4, bvp4c verification), identification of diffusion-limited and consumption-limited regimes, and the use of Bayesian-ridge regression for rapid estimation of parameter space by predicting the concentration at the center [78].

In the process context, it was emphasized that harsh crosslinking (chemicals, temperature, UV) may reduce viability, and thermosensitive bioink gelling at 37 °C without additional post-processing was described as an alternative; it was also indicated that a slower deposition rate of 30 µL/min and a soft chitosan substrate improved viability, and after printing, >70% viability was reported to be maintained up to day 5 in HepG2 and HUVEC co-cultures, as well as the effect of a 2:1 cell ratio (HUVEC:HepG2) on urea production with reference to the physiological level and stability up to day 7 [79]. It was pointed out that the oxygen profile strongly depends on the height of the medium column, the dimensions of the aggregates and the OCR, with recommendations of 1.1–1.3 mm of the medium height and ~3% of the seeding surface in the analyzed example of endocrine aggregates and pO_2_ thresholds related to the function and Michaelis–Menten parameters (Km ~0.4 mmHg, anoxia ~0.1 mmHg, secretion impairment ~2.5 mmHg), with FEM indicated as a tool for designing culture conditions and waste reduction [80]. It was also emphasized that although oxygen consumption can be described by Michaelis–Menten kinetics, reaction parameters and average OCR are not universal and depend on the cell density in 3D, and fitting oxygen profiles over time to the reaction–diffusion model (after reducing the geometry to 1D and unambiguously defining the boundary/initial conditions) requires caution in the interpretation of OCR (average vs. local, 2D vs. 3D) [81].

In reviews of cell damage in bioprinting, high shear stresses and nutrient deficiency in the construct were indicated as reproducible sources of viability decline, and CFD was presented as a method for quantifying shear stresses with the introduction of cell damage ratio indicators; in this context, inkjet limitations were also cited (resolution up to 50 µm, viscosity 3–30 mPa s, max. ~10^6^ cells/mL) and dispensing pressure was indicated as the dominant damage factor compared to nozzle diameter, with a parabolic trend of increasing shear stress and decreasing viability with increasing pressure [82]. In bioinert, thermosensitive hydrogels, a decrease in viability up to ~50% was reported 24 h after printing as a trend independent of initial conditions, interpreted mainly as an effect of malnutrition (lack of excess medium) and anoikis (lack of cell–ECM contact and adhesion sites), with the Mix control maintaining >80% viability after 4 h; in parallel, an increase in the expression of mechanoresponsive genes FOS and PTGS2 was observed with the contribution of gel incubation separated from the effect of printing itself (2–4 h), as well as differences in viability after printing depending on the diameter 22 G vs. 30 G and a trade-off between printability and short-term bioinks in comparison of Pluronic and POx/POzi bioinks [14].

Based on the literature review, no work was identified that combines hydrogel mechanics, oxygen diffusion and nutrient transport limitations, cross-linking kinetics, and biological determinants of cell survival in extrusion bioprinting in a single, coherent model. This indicates that the integration of these phenomena within a unified computational and design framework remains underdeveloped, and its systematic approach could represent a novel research direction.

AI-specific limitations include bias due to limited training data, limited interpretability of black-box predictions, and potential challenges in generalizing models to different hydrogel chemistries. To minimize these threats, we propose the use of physics-based neural networks that leverage mechanistic insights to improve robustness and interpretability. Additionally, active learning strategies will be implemented to prioritize data collection in regions of high uncertainty, improving model generalization. Explainability will be ensured by the use of eXplainable AI mechanisms such as SHAP. These additions will ensure balanced consideration of constraints along with specific strategies for increasing AI robustness and reliability.

A relevant recent experimental case is provided by Wales et al. [83], who investigated extrusion-based 3D printing of POMaC/PEGDA copolymers and optimized the process through an iterative trial-and-error procedure involving extrusion pressure, print speed, UV intensity, and UV exposure time. Importantly, the study also quantified printability by comparing measured cross-sectional dimensions of printed structures with the ideal STL geometry and related the formulation and process conditions to mechanical, adhesive, degradation, and biocompatibility outcomes. Although this work was not AI-driven, it clearly illustrates the type of coupled process–material–performance relationships that could serve as a valuable basis for future AI-assisted modeling and parameter optimization in bioprinting.

## 3. Research Gap and Problem Definition

Despite significant progress in modeling and optimization of individual stages of 3D bioprinting, current knowledge remains fragmented across distinct research areas and has not yet led to the development of a coherent set of tools supporting the entire process of designing a hydrogel–cellular construct. Literature analysis indicates an extensive knowledge base regarding the influence of extrusion parameters on cell viability and mechanical stress [1,2,3,4,5,6,7,8], cell residence time in non-crosslinked bioink [9,10,11,12,13], mechanisms of cell death in bioinert hydrogels [14,15,16,17,18], transport constraints related to oxygen diffusion and construct thickness [19,20,21,22,23,24,25,26,27,84], as well as strategies for reducing hypoxia through microcanalization, perfusion, and vascularization [28,29,30,31,32,33]. In parallel, models describing the influence of cross-linking strategies on the mechanics and cellular microenvironment [10,34,35,36,37], the relationship between gelation kinetics and shape fidelity [38,39,40,41,42,43], as well as computational tools for the analysis of transport, mechanics and behavior of the construct [46,47,48,49,50,51,52,53,54,55] were developed. The observed research gap is not due to a lack of data, but rather to the lack of their integration into a practical, coherent toolkit that could support the construct design process before bioprinting begins. While partially integrated approaches appear in selected studies, they typically cover only selected process components without simultaneously considering hydrogel mechanics, mass transport, fixation kinetics, and cell survival in a single computational framework [6,70,71,72,73]. This means that construct design is still primarily performed by sequentially combining results from different knowledge sources, rather than by utilizing an integrated toolkit capable of predicting interconnected material, process, and biological consequences.

From a research practice perspective, this leads to a significant limitation: even an extensive literature review does not provide the designer with a toolkit that would allow them to simultaneously assess whether a given geometry will be printable, whether the bioink used will maintain stability after deposition, whether the cell exposure time to preprinting conditions will remain within biologically safe limits, and whether areas of critical hypoxia will not appear in the finished structure. As a result, bioprinting optimization still relies heavily on trial and error, even though the cost of a single unsuccessful iteration in this area is exceptionally high. This applies not only to the consumption of expensive hydrogels, reagents, and instrument time but also to the loss of valuable biological material, including cultured cells, whose preparation requires time, resources, and properly conducted laboratory procedures. In this sense, the lack of a coherent predictive framework has not only technological and economic dimensions but also organizational and ethical ones, as it increases the number of experiments whose results could have been previously narrowed down computationally.

The key research problem currently lies not in the lack of partial models, but in the inability to use them together in a single decision-making process. In practice, this means that the designer does not have an approach that allows for simultaneous assessment of the impact of bioink selection, extrusion parameters, cross-linking strategy, and construct geometry on post-deposition stability, mechanical conditions in the material, and biological safety of cells [9,10,11,12,13,19,20,21,22,23,24,25,26,27,34,35,36,37,38,39,40,41,42,43,44,45,83]. Consequently, it is still impossible to reliably narrow down the design space before printing, even though it is at this stage that decisions crucial to the success of the process are made.

**Figure 1:**
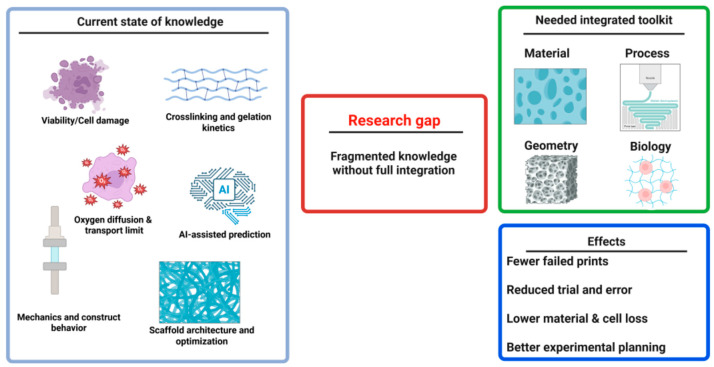
Schematic representation of the current state of knowledge, research gap, and the need to develop an integrated toolkit for designing hydrogel–cell constructs in 3D bioprinting.

Insufficient integration also applies to the computational layer. Models of transport, mechanics, and construct behavior, as well as AI/ML methods, have high cognitive value, but most often support only selected aspects of the process, without creating a coherent design environment enabling data exchange and joint evaluation of results [46,47,48,49,50,51,52,53,54,55,56,57,58,59,60,61,62,63,64,65,66,67,68,69,74,75,85,86]. Therefore, the research problem can be defined as the need to develop a coherent, potentially modular, design-before-print toolkit that will enable the simultaneous assessment of material, process, geometry, and biological constraints prior to experimental validation. A schematic representation of the current state of knowledge, the identified research gap, and the need to develop such a toolkit is presented in Figure 1.

## 4. Proposed Model and Technological Implications

To address the identified research gap, the proposed approach should be understood not as a single model, but as a coherent, modular toolkit supporting the design of hydrogel–cell constructs prior to experimental validation. This proposal stems from both the team’s own experience and a literature review, which indicates that despite significant progress in modeling selected aspects of bioprinting, there is still a lack of a single framework capable of simultaneously considering hydrogel mechanics, mass transport, fixation kinetics, and biological constraints related to cell viability [6,70,71,72,73]. Consequently, construct design is still primarily based on the sequential combination of results from separate areas of knowledge, rather than on an integrated approach enabling advance prediction of interconnected material, geometric, process, and biological consequences.

We define the AI framework as a hybrid system combining surrogate models and physically based neural networks (PINNs) to capture both data-driven and mechanistic aspects of the hydrogel–cell composite’s behavior. The main inputs for these models are bioprinting parameters (e.g., extrusion pressure, nozzle diameter, printing speed), material properties (e.g., viscosity, cross-link density), and cell-related variables (e.g., density, viability), while the outputs focus on predicted biomechanical properties and functional performance metrics. The training process involves the use of supervised learning on experimentally derived datasets, supplemented with synthetic data generated from numerical simulations. To ensure robustness, the dataset must integrate rheological measurements, mechanical test results, and structural features derived from imaging. Model performance is assessed using standard regression metrics (e.g., RMSE, R^2^) along with cross-validation and external validation on independent batches. Experimental data will be incorporated via co-learning or knowledge transfer strategies to improve generalization in data-poor settings. The workflow encompasses the interaction between experimental data acquisition, computational modeling, and AI-based prediction within the proposed framework.

The starting point for the proposed model should be a given target geometry, initially understood as a solid structure without pores and channels, delivered as a three-dimensional CAD model, e.g., in .stl, .obj, or .step format. This means that the user would primarily define the shape and functional purpose of the final construct, while the system would not treat this geometry as a final solution but as input data requiring further analysis. This analysis would have to include both geometric stability during deposition and biological constraints resulting from oxygen and nutrient transport, as the previous sections of the review indicate that construct thickness, local diffusion distances, and lack of adequate microchannelization can lead to the formation of critical hypoxic zones and reduced cell viability [19,20,21,22,23,24,25,26,27,28,29,30,31,32,33,84]. In this approach, the geometry could not be assessed solely in terms of its compliance with the spatial model but would also have to be interpreted in terms of its biological and technological feasibility [85].

**Figure 2:**
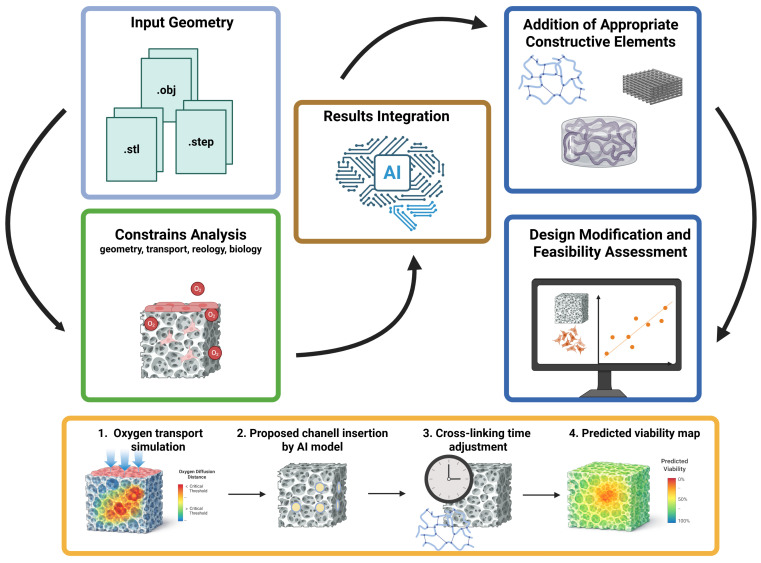
Conceptual diagram of an iterative design workflow including input geometry, constraint analysis, AI integration of results, design modification, and assessment of its feasibility in 3D bioprinting.

The fundamental logic of the proposed model would therefore involve transforming the target geometry into one that can be produced and maintained under 3D bioprinting conditions. In practice, this would mean assessing whether a given construct can be printed without losing structural integrity, whether its local architecture does not lead to exceeding critical diffusion distances, and whether it is necessary to introduce additional functional elements, such as diffusion channels or simplified microchannel systems. If necessary, the system could also propose the addition of appropriate supports or other structural elements to increase the stability of the manufacturing process. At the same time, the model should consider the impact of the cross-linking strategy and the rheological properties of the bioink on shape fidelity, post-deposition stability, and the mechanical conditions to which the cells are subjected during and immediately after printing [10,34,35,36,37,38,39,40,41,42,43]. As a result, the system would not only be used to reproduce a given solid, but also to modify it in a controlled manner, in accordance with constraints derived from the literature and adopted design criteria. The proposed iterative workflow, leading from input geometry through constraint analysis and result integration, to design modification and feasibility assessment, is presented in Figure 2.

A simple illustrative scenario of the proposed workflow may be described as follows: (i) the system first analyzes a solid target construct with respect to local diffusion distances and flags regions in which transport limitations may compromise biological feasibility; (ii) if such regions are identified, the design may be conditionally modified through the introduction of diffusion channels or supporting features; (iii) the modified geometry is then re-evaluated in terms of cross-linking conditions and structural stability; and (iv) the final output may include a biologically oriented feasibility assessment, for example in the form of a predicted viability distribution within the construct. In this sense, the workflow is intended not only to assess a given geometry but also to iteratively transform it into a biologically and technologically feasible design-before-print solution.

AI would play a special role in the proposed toolkit, not as a substitute for physical models, but as an integrating and predictive layer. The review shows that AI/ML is already being used to reduce trial-and-error, determine design space, map CMA and CPP to CQA, predict print quality, and support iterative optimization of process parameters [58,59,60,61,62]. At the same time, it was pointed out that AI can act as a systemic link connecting the design of bioink, geometry, printing parameters, and construct curing conditions, as well as support workflows covering the pre-printing stage, printing itself, and post-print quality assessment [59,69]. In the proposed approach, the AI layer could be responsible for integrating results from transport, mechanics, rheology, and process time models, identifying patterns difficult to capture in single-parameter analysis, and predictively assessing whether a given geometry variant and process conditions are within acceptable biological and technological limits. In practical terms, this AI layer may include surrogate models for parameter prediction and optimization, data-integration models for heterogeneous simulation and process outputs, and decision-support components operating under jointly evaluated technical and biological constraints.

Such a model would be closer to a design decision support environment than a classic numerical solver. Physical and simulation models would provide the computational basis for describing individual phenomena, while AI would integrate their results and support decision-making regarding geometry modification, process parameter selection, and reducing the risk of print failure. Considering time as a technological and biological variable would be particularly important here, as previous analysis has shown that the residence time of cells in uncrosslinked bioink cannot be treated as a neutral parameter, especially in the presence of additional factors that worsen survival conditions, such as dehydration, exposure to photoinitiators, or unfavorable environmental conditions [9,10,11,12,13]. This means that the proposed model should, in the future, enable not only the assessment of the construct’s printability itself, but also the prediction of whether the planned process will lead to exceeding the safe technological and biological window.

At this stage, this work focuses on the functional logic of the proposed model and the scope of problems that should be covered by a common design environment.

**Figure 3:**
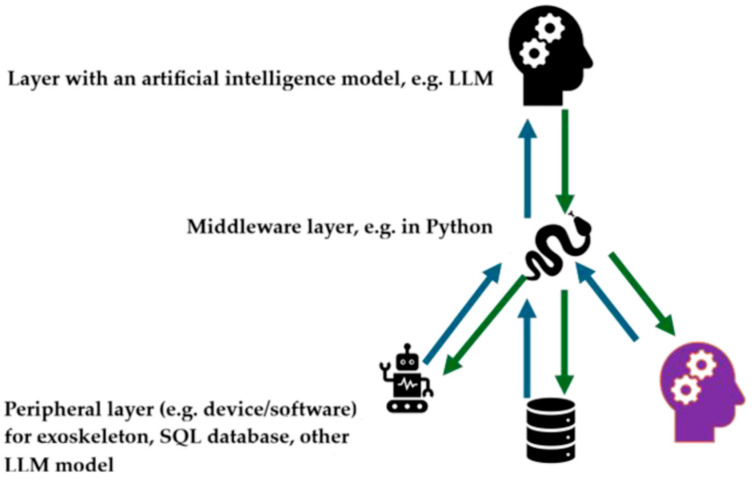
Conceptual framework for data integration, feedback, and AI decision support in a design-before-print environment.

The main component of the system is an AI model, for example, using large language models (LLM). A key role is played by the middleware layer, implemented in a selected programming language (e.g., Python), which serves as an integrating layer. This layer provides additional tools and functions to improve the AI model’s performance and manages communication with external modules. This architecture provides direct access to specialized databases (medical, physiotherapy, etc.) and enables interaction with other AI models and a dedicated reporting module. The conceptual framework for the interoperability of these components, with the AI layer playing a central role and enabling bidirectional information exchange between modules, is shown in Figure 3.

Sensor and imaging data from the prototype bioprinter will be preprocessed by a middleware layer, including noise filtering and feature extraction, and then fed into the AI training loop. Real-time data is used to update surrogate models or refine predictions using online learning strategies, allowing the system to dynamically adapt to observed discrepancies. Iterative feedback allows the AI layer to continuously optimize printing parameters while maintaining consistency with physical constraints. In this way, experimental feedback flows through the middleware to the AI models for training and fine-tuning.

LLMs are not intended for direct numerical prediction or physics-based modeling. We position LLMs primarily as an orchestration and interface layer that facilitates interaction between users and the underlying computational modules. Specifically, LLMs will be used for tasks such as assisting in experiment design, suggesting parameters based on prior knowledge, and structured searches of databases containing information on materials and bioprinting. They also play a vital role in knowledge retrieval and synthesis, enabling the integration of subject literature and previously generated results into the workflow. Importantly, all quantitative predictions and simulations remain the responsibility of dedicated ML models and physics-based solvers. We avoid overestimating the capabilities of LLMs and clearly define their role as auxiliary, not central.

The system will automatically generate microchannels or supports only when predicted diffusion distances exceed literature-defined thresholds (~100–200 µm), ensuring adequate nutrient and oxygen transport. The AI-based design module will consider functional and anatomical constraints while preserving key features of the original CAD model, such as external shape and key tissue-specific structures. Microchannel placement will be optimized for a balance between mechanical integrity and mass transport efficiency, based on surrogate models and physics-based constraints. This will allow for conditional insertion and constraint preservation.

The technological significance of the proposed approach lies primarily in shifting the burden of optimization from costly experimental trials to an earlier analytical and predictive stage. Instead of testing successive variants by trial and error, the designer could use an integrated simulation and AI environment to narrow down the design space early on, identify solutions with a high risk of failure, and select the most promising variants from the perspective of geometric stability, mass transfer, material properties, and biological safety. In this sense, the proposed model is not just another analytical tool, but an attempt to create a design framework in which geometric, mechanical, process, and biological requirements are assessed jointly, rather than as independent problems.

A validation plan that combines model predictions with experimental measurements obtained from a prototype bioprinter. We will define quantitative performance metrics, including RMSE, MAE, and R^2^, to assess the predictive accuracy of key biomechanical and functional outcomes. Model predictions will be systematically compared with both newly generated experimental data and benchmarks published in the literature to ensure external consistency. We will also incorporate cross-validation and holdout test sets to assess generalization performance under different bioprinting conditions. To reduce uncertainty, we will incorporate methods such as prediction intervals, Monte Carlo drop-out, or ensemble modeling to determine the robustness of the model results. Additionally, a sensitivity analysis will be performed to identify the most influential input parameters and assess model robustness. This will provide a comprehensive and rigorous framework for assessing the robustness of the proposed models.

Accordingly, the coupled optimization problem should be understood as a multi-objective task in which printability-, process-, mechanical-, and biology-related criteria are evaluated jointly rather than in isolation.

## 5. Future Research Directions and Conclusions

The concept of a modular design toolkit presented in the previous chapter requires further development in the form of a phased research program. Therefore, further work will focus on the gradual development and validation of the proposed framework, developed module by module, rather than as a single, complete system. This approach seems necessary from both a methodological and practical perspective, because each of the components considered—geometry, mass transport, rheology, fixation kinetics, process time, and cell viability—represents a separate research problem requiring separate experimental validation. Only after their independent development will it be possible to reliably integrate them into a single design environment supported by an AI layer.

The main novelty lies in the system-level integration strategy, specifically the unified coupling of physics-based models, data-driven AI components, and decision support mechanisms into a single workflow tailored to 3D bioprinting. Unlike existing frameworks that typically treat these elements in isolation, our approach emphasizes the dynamic interaction between modules, enabling iterative feedback between simulation, experimental data, and predictive modeling. An additional contribution is the inclusion of bioprinting-specific parameters and constraints in the optimization loop, which are often underrepresented in more general modeling frameworks. Furthermore, the decision support layer plays a key role in guiding parameter selection based on multiple criteria, such as mechanical performance and cell viability.

The first step in further research should be to develop tools for assessing the geometry and stability of the construct during bioprinting. In practice, this means determining whether the desired geometry can be printed and maintained without loss of structural integrity, collapse of subsequent layers, or uncontrolled deformation after deposition. At this stage, analysis of the shape, local load-bearing capacity of the deposited hydrogel–cellular structure, the sequence of material deposition, and the potential need for auxiliary elements such as supports or additional structural components will be crucial. In the initial phase of research, printing conducted directly “in air” should be the reference point, while strategies based on printing in a support bath or in existing hydrogel material should be considered a further, more advanced direction of development.

The next step will involve integrating transport constraints with the geometry generation logic. Based on previous analysis, it was determined that construct thickness, cell density, and the lack of appropriate microchannelization can lead to exceeding critical diffusion distances, the formation of hypoxic zones, and, consequently, cell death [19,20,21,22,23,24,25,26,27,28,29,30,31,32,33,84]. Therefore, the developed system should ultimately not only analyze the input geometry but also automatically propose modifications to the construct’s architecture that would limit the maximum diffusion distances. In practice, this primarily involves generating diffusion channels or simplified microchannel systems in those areas where solid geometry would be biologically unfeasible. It will be particularly important to maintain the functional purpose of the designed structure, so that elements responsible for mechanical or anatomical properties are not removed, but rather supplemented with solutions that improve mass transport conditions.

The next step will involve modeling the properties of the working mixture, comprising hydrogel, cells, and medium, and assessing the effect of this mixture on the process. Further research should therefore aim to quantitatively describe the rheology of bioink, the effect of cross-linking strategies on shape fidelity, and the mechanical conditions to which the cells are subjected during deposition and immediately after printing [10,34,35,36,37,38,39,40,41,42,43]. This is necessary because even a geometrically correct design may prove unfeasible if the mixture does not exhibit an appropriate printability window, post-deposition stability, or compatibility with the assumed fixation mechanism. In this sense, future design environments should gradually shift from analyzing geometry alone to analyzing geometry embedded in actual material and process properties.

A process time module, treated as a technological and biological variable, should be developed in parallel. Previous analysis has shown that cell residence time in uncrosslinked bioink is not a neutral parameter, especially in the presence of additional factors that worsen survival conditions, such as dehydration, exposure to photoinitiators, or unfavorable environmental conditions [9,10,11,12,13]. Therefore, the system should ultimately enable estimation of the duration of individual preparation and printing stages and indicate situations in which the planned process may lead to exceeding the safe technological and biological window. In practice, this could mean changing the printing strategy, dividing the process into shorter stages, replacing the bioink cartridge, or modifying environmental conditions during operation. From a biological perspective, time becomes not only an organizational parameter but also one of the factors directly affecting the final viability of the cellular material.

In the final model, we will employ a multiphysics coupling framework, including cross-linking kinetics, fluid flow, mass transport, and cellular response. The proposed framework utilizes a hybrid iterative coupling approach, where individual submodels are solved sequentially at each iteration step, simultaneously exchanging updated boundary conditions and state variables. This strategy enables stable integration of processes occurring at different temporal and spatial scales while maintaining computational feasibility. Information exchange between modules determines how outputs from one domain (e.g., shear stress from flow modeling) affect others (e.g., cell viability or cross-linking rate). The AI component complements this framework by approximating selected submodels or accelerating convergence in an iterative loop. These principles ensure that the coupling methodology is clearly defined, theoretically grounded, and transparent.

**Figure 4:**
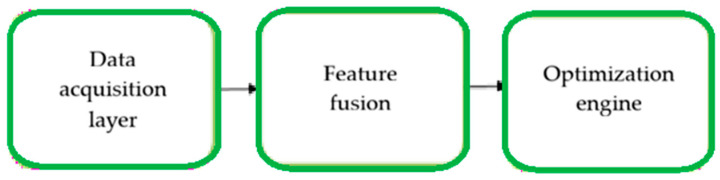
Prior design groundwork informing the future validation platform, rather than as a validated experimental system.

A key element of further research should also be the experimental validation of each of the developed modules. To this end, the company plans to develop its own prototype research bioprinter, designed not as a commercial device but as an experimental platform intended for data collection, process parameter control, and verification of the correctness of the developed models. This solution stems from the need for the most complete integration of measurement systems with the bioprinting process itself, which typical commercial devices do not provide to a sufficient extent. The designed printer will be based on a standard XYZ system, with a movable table in the Z axis and a movable head in the remaining axes. Expansion with additional sensors and modules for recording operating parameters is planned from the outset. As an illustration of the engineering groundwork underlying the planned validation platform, we include selected design documentation views of an earlier author-developed hydraulic bioprinting head. This material is presented as prior design work informing the future prototype platform, not as a validated experimental system (Figure 4). From the outset, the prototype will be designed for integration of pressure sensing in the hydraulic/extrusion circuit, piston-displacement monitoring, imaging of the deposition zone, and temperature monitoring of the printhead, bioink reservoir, and local working environment. Pressure and piston-position data will constitute the primary basis for estimating extrusion load and delivered bioink volume, whereas imaging data will be used to assess filament continuity, deposited-track width, and shape fidelity. Temperature measurements will support the interpretation of rheological behavior and process variability related to cross-linking and material stability.

Strategies to improve data availability and quality in the context of 3D bioprinting primarily include a standardized data schema encompassing key variables such as material properties, printing parameters, and biological outcomes to facilitate interoperability between studies. It is useful to use synthetic datasets generated from validated simulation models to supplement limited experimental data, particularly in underexplored parameter ranges. Furthermore, physics-based constraints can be leveraged to ensure that such synthetic data retain physical plausibility. Adopting reproducible experimental protocols and metadata reporting guidelines is recommended to improve the consistency and comparability of results. This includes the development of common repositories and collaborative platforms for data sharing within the scientific community and the development of transfer learning capabilities and data fusion techniques to leverage heterogeneous datasets from different sources.

The issue of particular importance in the planned prototype will be the use of an indirect piston drive with a hydraulic element, enabling more precise control of the volume and rate of bioink extrusion than in simple direct material feeding systems or pneumatic drive solutions. This solution is intended to not only enable more stable dosing of the hydrogel and cell mixture but also better link the mechanical parameters of the process with data used for model validation. In the initial phase of development, the prototype will be primarily adapted to printing directly in air, while more complex strategies, such as printing in a support environment or in previously deposited material, should be considered as a further development direction. A detailed description of the printer’s design, its technical architecture, and the applied measurement solutions will be presented in subsequent publications. However, in the context of this work, it is important to emphasize that this device is intended to serve as a research validation platform, supporting the development and calibration of the proposed toolkit. Planned calibration experiments will include: (i) calibration of pressure signals under controlled extrusion conditions, (ii) calibration of piston displacement against delivered volume and flow rate, (iii) camera-based calibration of deposited-track dimensions against reference measurements, and (iv) temperature calibration under controlled operating conditions. These measurements will provide synchronized process data for verification of process stability, comparison with simulation outputs, and future validation of AI-assisted predictive models.

At a later stage, the AI layer will become particularly important and should be developed not as a standalone substitute for physical models, but as a mechanism integrating simulation results and experimental data. This means that AI should be gradually trained on data from transport, mechanics, and rheology models, as well as from measurements obtained during experimental validation. In this approach, AI could support the identification of patterns invisible to single-parameter analysis, narrowing the design space, and multi-criteria design optimization, taking into account mechanical, process, and biological criteria simultaneously [58,59,60,61,62,69]. Of particular importance here will be the use of feedback between simulation and experiment, so that subsequent model iterations can be adjusted based on actual process data. Data obtained from the printer prototype can, in this approach, serve not only as validation material but also as a basis for further training and refinement of the AI layer.

AI techniques for integration and prediction include surrogate machine learning models such as feedforward neural networks and gradient-boosted regression trees, supplemented with physics-aware neural networks to ensure mechanistic consistency. Heterogeneous outputs from simulation modules—including mechanical, chemical, and cellular variables—are unified through feature normalization and embedding in a common vector space to ensure compatibility with AI models. The middleware handles this preprocessing, aggregating the outputs, applying scaling or encoding as needed, and formatting them for predictive models. AI models are trained on the combined experimental and simulation data to maintain predictive accuracy across modalities. This makes the role and operation of the middleware transparent and technically justifiable.

From a computer science perspective, the current state of AI-assisted 3D bioprinting of hydrogel–cell composites has seen rapid growth since 2020, driven by the need to model the complex interplay between material composition, process parameters, and biological response [87,88]. ML models are now routinely used to predict the rheological and biomechanical properties (e.g., viscosity, elasticity, and viscoelasticity) of bioinks based on composition and shear conditions, achieving very high predictive accuracy and reducing experimental effort [89]. AI enables multiparameter optimization of printing conditions (nozzle diameter, pressure, velocity, and others) while limiting cell viability, often keeping stress levels within biological safety limits [90]. Hybrid approaches combining machine learning (ML) with physics-based models (e.g., finite element analysis and rheological laws) are increasingly being used in biomechanical modeling to capture cell–matrix interactions and stress distribution in printed scaffolds. AI-powered inverse design frameworks allow researchers to define target functional parameters (e.g., stiffness, porosity, or degradation rate) and automatically generate optimal hydrogel formulations and printing strategies [91]. Closed-loop and real-time control systems are emerging, often integrating computer vision and deep learning (DL), that monitor fiber formation, detect defects, and dynamically adjust printing parameters during fabrication. Data-driven multi-objective optimization expands the available design space for hydrogel composites, enabling simultaneous tuning of mechanical strength, biocompatibility, and print fidelity beyond traditional trial-and-error methods. A key limitation remains the scarcity and heterogeneity of high-quality datasets, which can limit model generalization and lead to unreliable predictions outside the trained domains. Future perspectives emphasize the integration of autonomous laboratories, ML, and generative AI (genAI) for autonomous experimental design, bioink optimization, and the discovery of novel hydrogel–cell composite systems, as well as the use of tissue digital twins (DTs) [92]. AI-assisted modeling of biomechanical and functional parameters in bioprinted hydrogel–cell systems is moving toward fully adaptive, data-driven workflows, but their clinical implementation still requires validation, standardization, and regulatory approval.

Chronic and complex wounds remain a significant clinical challenge due to the complex structure of, e.g., skin and multifactorial healing processes. Traditional treatments often fail to fully restore the tissue’s natural structure and functionality [93]. Recent advances in 3D and 4D bioprinting have revolutionized wound management, enabling the fabrication of tailored, biomimetic skin substitutes that improve healing outcomes [94,95,96,97]. The complexity of the skin and the subsequent stages of repair have been elucidated while identifying the shortcomings of existing therapies [98,99]. The evolution of 3D bioprinting from basic additive manufacturing to advanced techniques enabling the precise placement of biomaterials and cells for skin regeneration is also explored [100,101]. Particular attention is given to bioinks, including natural polymers, synthetic hydrogels, decellularized extracellular matrices, and composite systems designed to replicate natural tissue properties [102,103]. Furthermore, 4D bioprinting introduces intelligent, stimuli-responsive materials that can dynamically adapt to changing wound conditions [104,105]. Current scientific and clinical discussions include key cellular components, multi-layer bioprinting strategies, and personalized solutions such as in situ wearable devices and AI-assisted manufacturing [106,107]. Beyond biomedical applications, 3D printing integrated with AI, IoT, and cloud computing systems is transforming infrastructure and manufacturing, enabling efficient, customizable, and sustainable production [108,109]. Despite these advances, challenges remain related to scalability, regulation, quality control, and integration with new AI-based and hybrid platforms, underscoring the need for further research and adaptation to recent advances such as digital twins and closed-loop bioprinting systems [110,111].

The proposed direction for further research aims to develop a coherent, step-by-step set of tools to support bioprinting design before the experiment. Its ultimate value will not only be the ability to analyze individual phenomena but also the integration of geometric, transport, mechanical, rheological, and biological requirements into a single decision-making process. Of particular importance here will be the combination of simulation models with a proprietary experimental platform and an AI layer developed based on real-world data, which in the future may enable more accurate predictions of construct feasibility and process flow. Such an environment could reduce the number of costly experimental trials, improve the predictability of 3D bioprinting, and create a foundation for a more rational and, at the same time, more ethical design of hydrogel–cell constructs. In this sense, the presented concept does not address the issue but rather provides a direction for further work on design-before-print tools, the lack of which was clearly identified in the literature review [6,69,70,71,72].

## Data Availability

No new data were created. Data sharing is not applicable to this article.

## Abbreviations

The following abbreviations are used in this manuscript:

AI	Artificial intelligence
DL	Deep learning
genAI	Generative artificial intelligence
ML	Machine learning
